# Laser and LIDAR in a System for Visibility Distance Estimation in Fog Conditions

**DOI:** 10.3390/s20216322

**Published:** 2020-11-05

**Authors:** Razvan-Catalin Miclea, Ciprian Dughir, Florin Alexa, Florin Sandru, Ioan Silea

**Affiliations:** 1 Automation and Applied Informatics Department, Politehnica University Timisoara, 300006 Timisoara, Romania; miclea_razvan@yahoo.com (R.-C.M.); florin.d.sandru@gmail.com (F.S.); 2 Communication Department, Politehnica University Timisoara, 300006 Timisoara, Romania; ciprian.dughir@upt.ro (C.D.); florin.alexa@upt.ro (F.A.)

**Keywords:** laser, LIDAR, fog, visibility distance, visual acuity, autonomous vehicle

## Abstract

Visibility is a critical factor for transportation, even if we refer to air, water, or ground transportation. The biggest trend in the automotive industry is autonomous driving, the number of autonomous vehicles will increase exponentially, prompting changes in the industry and user segment. Unfortunately, these vehicles still have some drawbacks and one, always in attention and topical, will be treated in this paper—visibility distance issue in bad weather conditions, particularly in fog. The way and the speed with which vehicles will determine objects, obstacles, pedestrians, or traffic signs, especially in bad visibility, will determine how the vehicle will behave. In this paper, a new experimental set up is featured, for analyzing the effect of the fog when the laser and LIDAR (Light Detection And Ranging) radiation are used in visibility distance estimation on public roads. While using our experimental set up, in the laboratory, the information offered by these measurement systems (laser and LIDAR) are evaluated and compared with results offered by human observers in the same fog conditions. The goal is to validate and unitarily apply the results regarding visibility distance, based on information arrives from different systems that are able to estimate this parameter (in foggy weather conditions). Finally, will be notifying the drivers in case of unexpected situations. It is a combination of stationary and of moving systems. The stationary system will be installed on highways or express roads in areas prone to fog, while the moving systems are, or can be, directly installed on the vehicles (autonomous but also non-autonomous).

## 1. Introduction

Researches in the field of visibility started a few decades ago and are still of great interest and high importance. The most relevant works that are related to this topic were published by Middleton [1] and McCartney [2] and were continued by Narasimhan et al. [3]. This work focuses on the automotive field and the great importance of visibility on public roads. Statistics confirm that, during the last decades, road traffic injuries are one of the main factors of death worldwide [4], and bad weather conditions, associated with speed are considered one of the major issues [5]. According to the U.S. Department of Transportation [5], fog/rain/snow is considered to be in the top five environment-related critical reasons. This issue becomes more important now when autonomous vehicles are rolling out on public ways. There are already some examples when the autonomous vehicle failed due to visibility problems: autonomous vehicle developed by Google failed the tests in bad weather conditions [6], while Tesla had, in 2016, the first fatal accident when neither the vehicle system nor the driver observed a truck on the highway [7].

The expectation from the autonomous vehicle would be to significantly decrease the number of accidents and, of course, the number of lost lives on public roads; according to the U.S. Department of Transportation, fully automated cars can reduce traffic fatalities up to 94% [8]. That is why the biggest automotive companies around the world are working to develop reliable and robust systems that can fulfill the expectations. In the area of visibility improvement, they started to redesign the headlight, by using new technologies like led or laser which came with benefits like better directivity or emitted light closer to the natural one [9], but they also introduced new features, one example is auto-dimming technology [10]. These improvements were useful during night conditions but for foggy conditions, the problem remains unsolved. Accordingly, the methods and systems that are available in the scientific literature related to this issue can be split into two categories: visibility enhancement systems and fog detection (and visibility distance estimation) systems. Approaches from the first category are mainly based on image processing, by dehazing the input images taken with a camera, detecting pedestrians, traffic signs, or other obstacles from the road. Different methods were used for these approaches, like Koschmieder law-based methods, dark channel prior, image segmentation, contrast enhancement, etc. For the second category, which we called fog detection and visibility distance estimation, prevail methods that are based on optical power analysis but are also considered image processing methods. Approaches based on optical power analysis can be split into direct transmission and back-scattering. For the first subcategory, an optical signal sent by a source is evaluated with an optical receiver, after passing an area with fog particles. For the second one, the reflected optical signal is evaluated, the shape of the signal being modified after hitting a “wall” of fog particles. LIDAR (Light Detection And Ranging) system works also on the back-scattering principle, but, in this case, it is measured the time of flight, from the moment of sending the signal until it returns into the receiver. In literature are also presented radar-based approaches [11] for bad weather detection. The radar is working on the same principles as the lidar does, but it uses radio waves instead of optical ones. Related to the image processing part for fog detection, most of the methods from the literature are based on global image analysis.

All of the above-described approaches, from both categories, have their advantages and disadvantages. To obtain better results and build a robust and reliable system, automotive developers started to combine them, in order to carry out the weak points of a sub-system through another sub-system. After five years of research in this field (studying deeply the methods from the literature) and also considering the big amount of experiments realized in the laboratory, our goal is to approach a collaboration between systems that can estimate the visibility distance. Additional, having in mind the idea of creating a generic system, which can be used as stationary (on highways, airports, etc.) or moving system (inside the vehicle or even on autonomous vehicle), we analyzed the link between the results offered by two measurement systems (laser and LIDAR) and the human visual acuity, which is also affected by the fog.

The paper is structured, as follows: Section 2 presents approaches that are find in related work to the two broad categories—fog detection and visibility distance estimation. Section 3 presents the laboratory experimental setup used to test methods from visibility enhancement, fog detection and visibility distance estimation categories. The focus of this paper will be on fog detection methods. In Section 4, the experimental results are presented and discussed. In the last section, Section 5 reports the conclusions together with the proper collaborative system to be used for fog detection.

## 2. Related Work

Fog is a meteorological event [12] that has a bad impact on visibility. The visibility range is reduced under 1 km and this happens due to the atmospheric particles that absorb and scatter light; based on the particle size and concentration, visibility can be reduced from slight opacity to zero.

For the transportation field, on public roads, the area of interest to be evaluated from the visibility point of view is 300 m; if the drivers are notified about the hazards or risk in this area, the time to take actions and avoid a possible accident is short (it remains around four seconds for a reaction at a speed of 130 km/h), so the system needs to process all of the data in real-time. During the last two decades, there were plenty of methods presented, which can basically be split into two broad categories: visibility enhancement and fog detection.

In the first category, the methods are based on image processing; one of the most known and used law in this field is the Koschmieder law [13], which is the starting point for most of the methods from the image dehazing field. Going deeper into the scientific literature, there are various approaches that are related to haze removal and visibility improvement, which can basically be split in two subcategories: single input images and multiple input images.

Recently, researches have focused more on image dehazing methods that use a single image as input [14], because multiple numbers of frames that need to be treated to obtain a non-foggy image results in higher costs and time in processing the data. We are not deepening this direction now, here.

Related to the second category, fog detection and visibility estimation, we can identify methods that are based on optical power measurements but also image processing. Narasimhan presents one of the most relevant work [3], paper based on the reputed researches of Middleton [1] and McCartney [2].

In the optical power measurements category, there can be listed the following two approaches:

(i) Direct transmission measurements, which consists in measuring the optical power of a light beam after passing a fog layer.

In paper [15], the authors present a laboratory experimental approach that analyzes the fog influence on different optical sources, like led and laser. This work is a good starting point for differentiating fog in several classes. In [16], Pesek et al. present an outdoor conditions fog density detection approach, based on free-space optical link attenuation. The work is continued in [17,18] by Brazda et al. which derived a formula, between the optical link attenuation (β) and visibility (V), from the Beer–Lambert law:

(1) $$\beta =3.91/V\;[km^{-1}]$$

According to the International Commission on Illumination (CIE) definition (845-11-21), the visibility is established from a contrast threshold of 5%, and based on this statement, many studies indicate the relation between optical attenuation and visibility as:

(2) $$\beta =3/V\;[km^{-1}]$$

(ii) Backscattering measurements consist of measuring the reflected/scattered light from a fog layer.

Lidar systems can be mentioned in this category. By measuring the return time of the reflected light in the receiver (also called time of flight), can be calculated the visibility distance (the distance to the fog layer) [19]. Other approaches using lidar systems can differentiate fog conditions from other objects by analyzing the shape of the responses ( [20,21]). On the same principle, of sending and receiving short infrared impulses, is based the system that was proposed by Ovsenik et al. in [22]; the system measures the liquid water content from the environment and by comparing the values with some predefined thresholds, it can determine the presence of fog.

The image processing methods from this category are mainly based on image global features analysis. The approaches have the main goal of classifying the images into different categories (non-fog, low/medium/dense fog) by analyzing their features. In [23], Alami et al. considered saturation and correlation between the three RGB channels, as main parameters for fog detection. In [24], it was analyzed the power spectrum of the input image to detect possible fog conditions. The power spectrum is the squared magnitude of the Fourier transform of the image and contains information related to the frequencies from an image. Spinneker et al. propose in [25] a method that detects fog by considering the frequency distribution of an input image; the idea of this proposed method is that the noise areas result in high frequencies. Zhang et al. present in [26] a comparison between several histogram evaluation methods. In [27], direct adjustments to the LIDAR parameters were made in order to improve the data quality.

In our experiments, we treated fog detection (and visibility estimation) going further in a deep analysis of the fog structure. The laboratory setup was built in such a way to offer the possibility of simulating the outdoor conditions, to repeat them several times, and to conclude which are the advantages and disadvantages of every method.

## 3. Methods and Materials

The detection and study of fog phenomena are based on a physical-mathematical theory. This section will show the main physical-mathematical methods that were related to fog detection through light scattering (Rayleight scattering and Mie scattering). These are the main theories used in explaining the effects of fog, in understanding and interpreting the results.

### 3.1. Rayleigh Scattering

Rayleigh scattering [28] is applicable for light scattering, where the size of the particles (*x*) from the atmosphere is much smaller than the wavelength (λ) of the light (*x* < λ/10). The intensity of the scattered radiation, *I*, can be defined as the product between the initial light intensity $I_{0}$ and the Rayleigh scattering term *S* (λ, θ, *h*):

(3) $$I=I_{0}S\left(\lambda ,\theta ,h\right)=I_{0}\frac{\pi^{2}{\left(n^{2}-1\right)}^{2}}{2}\frac{\rho \left(h\right)}{N}\frac{1}{\lambda^{4}}\left(1+\cos^{2}\theta \right)$$

where λ is the wavelength of the input light, θ is the scattering angle, *h* is the position of the point, *n* is the refraction index, *N* is the density of the molecular number of the atmosphere, ρ is the density level that is equal to 1 at sea level and decreases exponentially with *h*. The Rayleigh scattering equation shows how much light is scattered in a specific direction, but it does not indicate how much energy is scattered overall. For this, it must be taken into consideration the energy scattered in all directions:

(4) $$\beta \left(\lambda ,h\right)=\frac{8\pi^{3}{\left(n^{2}-1\right)}^{2}}{3}\frac{\rho \left(h\right)}{N}\frac{1}{\lambda^{4}}$$

where β (λ, *h*) represents the fraction of energy lost after a collision with a single particle. This is known as the Rayleigh scattering coefficient or the extinction coefficient.

The initial Equation (4) that described Rayleigh scattering can be rewritten, as follows:

(5) $$S\left(\lambda ,\theta ,h\right)=\beta \left(\lambda ,h\right)\gamma \left(\theta \right)\to \gamma \left(\theta \right)=\frac{s\left(\lambda ,\theta ,h\right)}{\beta \left(\lambda ,h\right)}=\frac{3}{16\pi }\left(1+\cos^{2}\theta \right)$$

where the first term β (λ, *h*) controls the intensity of the scattering while the second one γ (θ), the direction of scattering. The last term is not dependent on the input light wavelength anymore.

The Rayleigh scattering is applicable for particles whose size is less than 10% from the incident radiation wavelength. If the particle size becomes bigger than this value, the Mie scattering model can be applied in order to identify the intensity of the scattered light, which is the sum of an infinite series of terms, not just a simple mathematical expression, like in the Rayleigh model.

### 3.2. Mie Scattering

According to ISO 13321:2009, the model is applicable for particles under 50 μm. Dust, pollen, smoke, and microscopic water drops that form mist or clouds are common causes of Mie scattering. When comparing to Rayleigh scattering, for Mie scattering the influence of input light wavelength is very low; the variation of the intensity is much stronger in the front direction comparing to the rear one, and the difference increases with the particle dimension. Mie theory is a theory of the absorption and scattering of flat electromagnetic waves of uniform isotropic particles, having simple shapes (sphere, infinite cylinder) that are part of an infinite, dielectric, uniform, and isotropic environment.

The main scope of the theory is the calculation of efficiency coefficients for absorption ($Q_{a}$), scattering ($Q_{s}$), and extinction ($Q_{e}$), which are connected through the following formula [25]:

(6) $$Q_{e}=Q_{a}+Q_{s}$$

The scattering and extinction coefficients can be represented as infinite series, but, for satisfactory convergence, the series shall not be longer than $j_{max}$ = *x* + 4 × 1/3 + 2 (where *x* = 2πr/λ ) is the diffraction parameter):

(7) $$Q_{s}=\frac{2}{x^{2}}\sum_{j=1}^{\infty }\left(2j+1\right)\left({\left|aj\right|}^{2}+{\left|bj\right|}^{2}\right)$$

(8) $$Q_{e}=\frac{2}{x^{2}}\sum_{j=1}^{\infty }\left(2j+1\right)Re\left\{aj+bj\right\}$$

Re is the real part of the complex numbers $a_{j}$ and $b_{j}$:

(9) $$a_{j}=\frac{\psi j\left(x\right)\left[\frac{\psi j^{\prime }\left(m_{\lambda }x\right)}{\psi j\left(m_{\lambda }x\right)}\right]-m_{\lambda }\psi j^{\prime }\left(x\right)}{\xi j\left(x\right)\left[\frac{\psi j^{\prime }\left(m_{\lambda }x\right)}{\psi j\left(m_{\lambda }x\right)}\right]-m_{\lambda }\xi j^{\prime }\left(x\right)};\;b_{j}=\frac{m_{\lambda }\psi j\left(x\right)\left[\frac{\psi j^{\prime }\left(m_{\lambda }x\right)}{\psi j\left(m_{\lambda }x\right)}\right]-\psi j^{\prime }\left(x\right)}{m_{\lambda }\xi j\left(x\right)\left[\frac{\psi j^{\prime }\left(m_{\lambda }x\right)}{\psi j\left(m_{\lambda }x\right)}\right]-\xi j^{\prime }\left(x\right)}$$

The two coefficients $a_{j}$ and $b_{j}$ are called the Mie coefficients or the expansion parameters; these are expressed in terms of Riccati–Bessel functions $\Psi {}_{j}$(*t*) and $\xi {}_{j}$(*t*), which, in their turn, are expressed as Bessel functions:

(10) $$\Psi_{j}\left(t\right)=\sqrt{\frac{\pi t}{2}}J_{j+1/2}\left(t\right);\xi_{j}\left(t\right)=\sqrt{\frac{\pi t}{2}}J_{j+1/2}\left(t\right)+{\left(-1\right)}^{ni}J_{-n-1/2}\left(t\right),i=\sqrt{-1}$$

The absorption coefficient $Q_{a}$ is determined based on the other two coefficients $Q_{e}$ and $Q_{s}$, while using (6).

**Figure 1:**
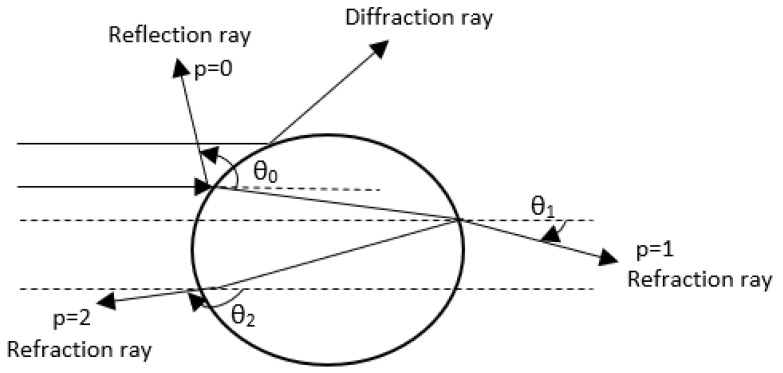
Scattering model for geometrical optics.

For particles that are much bigger than the wavelength of the incident light (at least 10 times bigger), are applicable the geometric optical laws. According to the geometrical optics, the amplitude of the scattered light is an overlapping of the reflected, refracted, and diffracted fractions. The assumption is that all incident rays coming from the ambient medium and crossing a particle are parallel. Each of these emerging rays is characterized by two parameters: the incident angle (θ) and number of chords that a ray makes inside the particle (p), as can be observed in Figure 1.

The incident rays are partly reflected and partly refracted several times when hit a particle surface, excepting the case of total reflection. In reality, the particle surface has irregular shape, so the rays can converge or diverge based on that. Beside these phenomena, the rays’ amplitude is also attenuated due to the effect of absorption.

For the experimental part, where the fog particles generated in the laboratory are studied and their impact on the incident light is analyzed, we will use Mie scattering (MiePlot tool) as long as it is proven (in [26]) that the agreement between geometrical optics scattering and Mie scattering is excellent, especially for larger particles.

### 3.3. Setup Used

The main goal of this research is to propose a reliable system (collaborative system), which is able to determine the visibility distance in bad weather conditions, and to help the driver by offering useful information. Of course, the next and natural step for this stage of research will be to install it on a highway and communicate directly with an vehicle, in order to validate the results in real fog conditions.

**Figure 2:**
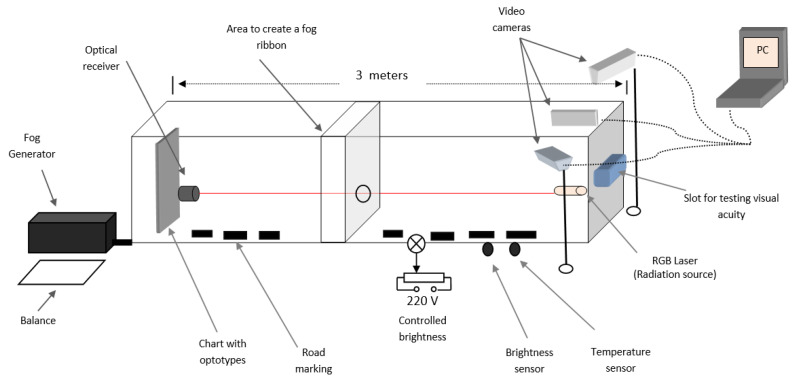
Experimental setup.

Before defining which is the proper system configuration for such a task, our research team had to evaluate and test the methods from the related work and to compare them. We needed to build up a generic laboratory setup (Figure 2) to be able to test different methods, from the literature, to find their advantages and disadvantages.

The main requirements for this setup are presented in continuing.

(a) Offer the possibility to realize different foggy conditions—for this, it is used a fog generator (FOG B-500), together with a balance (jewelry scale, for good accuracy), to measure the liquid spent for a specific level of fog; knowing the chamber volume it is easy to determine the fog density.

(b) Flexibility to test methods from both categories described above, visibility enhancement, and fog detection. Mandatory for both categories are the video cameras; the setup is equipped with cameras (Canon HF G10) inside the chamber, but also outside it, in order to monitor the entire process. The images acquired with the cameras are sent to a PC where they are processed. For the second category (fog detection), in the setup, there is an optical source (laser Lambda lll-2 HE-NE) and an optical receiver (Newport Power Meter Model 1918-c), which can be moved based on the method rhat you want to test (direct measurement or backscattering).

(c) Fulfill ophthalmologists’ requirements—the length of the chamber is three meters, to assure visual acuity evaluation requirements. During the experiments, in different fog conditions, the optotypes from the eye chart (44.3 mm biggest optotype and 4.43 mm smallest optotype from the chart) are monitored with a camera while using OCR (Optical Character Recognition) algorithms. On the other hand, in the same fog conditions, the visual acuity is evaluated by observers that need to read the optotypes by observing them on the “slot for testing visual acuity” (Figure 2—right side).

(d) The experimental setup shall offer the possibility, besides creating a uniform fog, to create a fog ribbon; the researches from the literature only consider fog density when analyzing the influence on optical power and visibility but fog thickness can be also extremely important. In the same fog conditions, more dispersed along the length, we can obtain different results as for a limited distance. On the other hand, we get the same results for fog dispersed in a more confined space with high density and a lower density in an extended space. The conclusion is that the laser beam is impacted by the number of particles encountered on its path. It allows for us to perform experiments at the laboratory level and extrapolate the resulting principles to real conditions.

### 3.4. Measurement Methodology

This subsection describes the way that the process takes place. The first step of our process is the write down all the preconditions, before starting the measurements: temperature, brightness and humidity inside the chamber; these parameters are very important when analyzing the fog impact.

Afterwards, the measurement process starts, fog is generated inside the chamber while the liquid quantity is monitored; after around 30 s (time need to have a uniform fog spread inside the chamber), we start to gather data from the (monitoring) tools: data from the optical power receiver are stored in a file, monitoring the fluctuation of the optical power source; data from the LIDAR are stored in another file, while for the telemeter, the data are gathered manually.

Related to the cameras, the one that evaluates the eye chart takes a frame at every second and stores it on the PC while the exterior one records the entire process. The images from the inside camera are processed while using an OCR algorithm, thus obtaining the optotypes that are still visible on the chart. Afterwards, the results are confirmed with the ones that were obtained from human observers, in the same foggy conditions.

The assessment time must be less than 5 min. for the fog conditions to be similar. After the monitoring process is over, the comparison between all of these results are performed manually.

## 4. Experiments and Results

This section presents the fog particles’ size influence on a light beam, first by analyzing the fog particles generated with our tool, FOG B-500 (Section 4.1); then, in Section 4.2, is presented a comparison between an automatic system (based on a laser device and an optical receiver) and human observers, related the visual acuity. Section 4.3 presents the fog impact on backscattering devices, while using a LIDAR and telemeter. The last Section 4.4 proposes a new safety system for visibility distance estimation.

### 4.1. The Influence of Fog Particles Size

We start our first experiments with the analysis of the fog particles, their structure, and density, for the fog generated in the laboratory setup. Fog is an accumulation of particles, of different sizes, whose diameters vary between tens to hundreds of microns. A fog cloud composed of smaller particles, but a higher density of particles, has, as a result, a more spoiled light signal after crossing such an area and, of course, lower visibility.

In [17] V. Brazda measured the attenuation of a light beam based on fog particles’ size distribution. The first parameter used in their research is the fog density, based on which visibility can be estimated. Other parameters monitored are the particle size and liquid water content, which are measured while using PVM-100 (Particle Volume Monitor).

**Figure 3:**
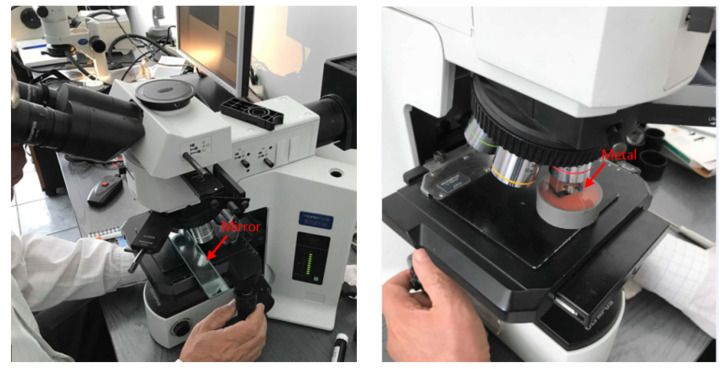
Fog particles analysis using Olympus BX51M microscope: on mirror (**left**) and on metal (**right**).

For our experiment fog, was generated with a fog generator (Fog B-500 model), which transforms a water-based liquid (the substance is a mixture of multi-valued alcohols and water, 2,2${}^{\prime }$-Oxydiethanol, Diethylene glycol (DEG), in a percentage <25%) into fog by warming it until it is vaporized and then splashing it out. Fog is generated at the contact of the splashed liquid with the colder air outside the machine. The fog particles were measured with a microscope, Olympus BX51M, by impressing the fog particles first on the mirror and then on a shiny metal (Figure 3), but we concluded that the deposit surface does not cause any difference. Following this hypothesis we were able to obtain a static distribution of the fog particles that could be analyzed (in the fog cloud the particles are of different sizes and the dynamics of particle variation is extremely large).

The entire experiment was monitored by a camera that was connected to the microscope, the images being acquired, displayed in real-time, and afterward analyzed while using a special application that was developed by Olympus, called analySIS. The microscope is capable of offering a zoom of ×500, which allows for us to observe, measure, and deep analyze the structure, size, shape, and density of the particles. The ROI (region of interest) from an image shall be first selected, an area where the tool can count the number of particles, but can also calculate the density of particles in that area. Related to the particle proprieties, the tool can measure the surface (projected area) of every particle, the shape factor, which is the ratio between the area of a certain particle and the mean area of the particles from the ROI, the aspect ratio, which is the ratio between the particle’s width and its height; after gathering all of the above-described parameters, the particles are framed in different categories.

**Figure 4:**
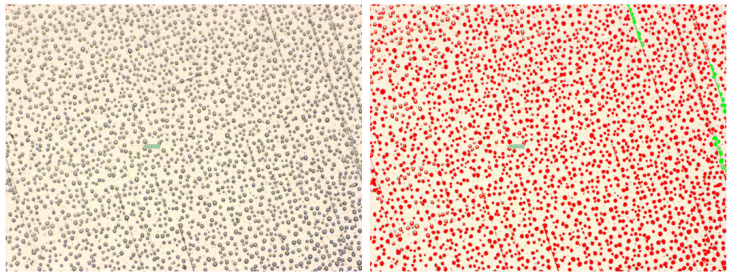
Fog particle analysis at ×50 zoom.

**Figure 5:**
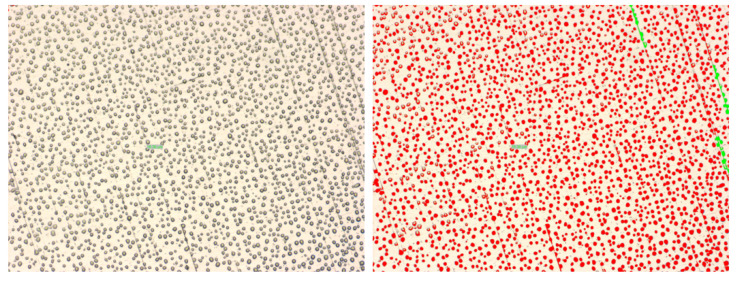
Fog particle analysis at ×200 zoom.

The figures below (Figure 4 and Figure 5) present two examples of particles analysis, first one at a zoom of ×50 and the second one at a zoom of ×200; the dimension of the particles varies between approximately 1 μm and 2300 μm, the rest of the parameters being noted below. Fog particle size is considered between 5 μm and 50 μm, so the maximum values do not correspond to fog droplets, but to raindrops.

In the region of interest described above (Figure 4), having an area of 2.3 ×$10^{6}$ μm${}^{2}$, are identified 2280 fog particles, which cover 19.68% from the image. The particles dimension vary between 23.32 μm and 2299.35 μm, the average for all 2280 particles being 199 μm.

For the second example (Figure 5), at a zoom of ×200, the ROI is more reduced when compared to the first one, having an area of 143.843 μm${}^{2}$, the total number of particles being 335, which cover 22.75% from the selected area. In this case, the particles’ dimensions varied between 1.45 μm and 946 μm, with an average of 98 μm.

The next step in our research is to mathematically determine the scattering and absorption influence of the particles on the laser beam, while using the Mie scattering approach. For this, we used the MiePlot tool (Version 4.6). Afterward, we will compare the mathematical results with the empirical ones, obtained in the experimental setup, related to the laser beam scattering and absorption. We choose to analyze the first scenario that is presented in this section, with the principles being the same for other cases.

**Figure 6:**
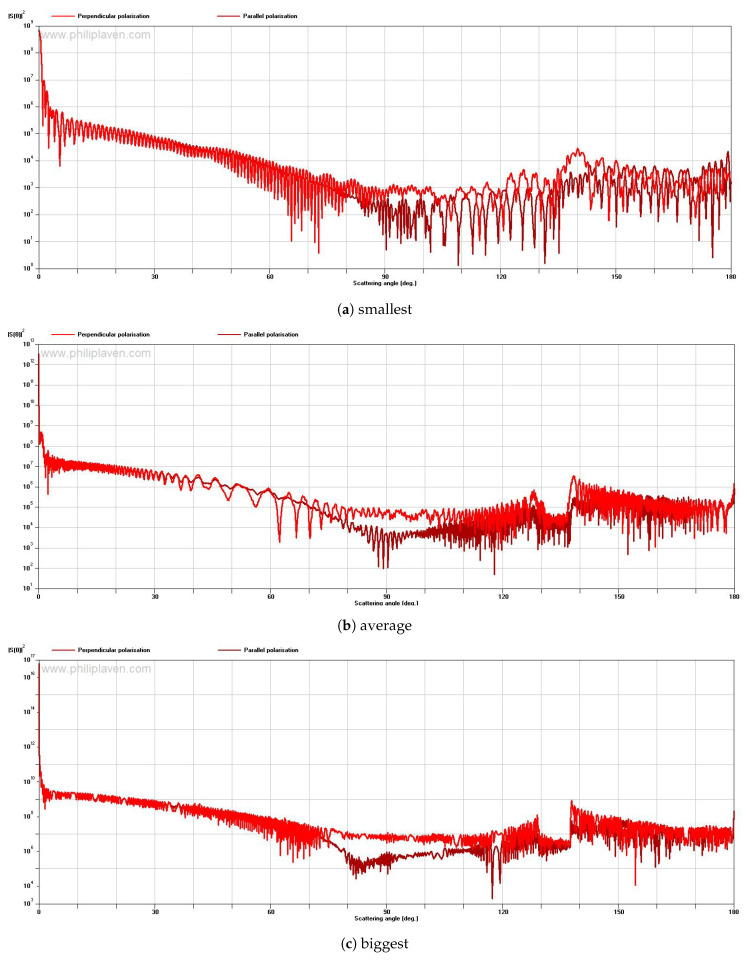
Scattering intensity vs scattering angle for (**a**) smallest, (**b**) average and (**c**) biggest particle

In the next figure (Figure 6), we analyzed the scattering impact caused by the smallest ( 23.32 μm), average ( 199 μm), and biggest ( 2299.35 μm) values of the particles from the first example presented. As mentioned above, the values that are bigger than 50 μm are not part of the fog particles category, but, in the experiment, we wanted to highlight the different particle’s influence on an optical source. It is analyzed the scattering intensity, S(θ), at different scattering angles, θ.

From the graphs that are presented in Figure 6, it can be observed that, with increasing particle size, the scattered intensity drops off increasingly strongly with increasing scattering angle. This confirms the theory that an environment composed of smaller particles and higher density has a bigger impact on light beams but also on visibility, when comparing to bigger particles. For all the three cases described above, the forward scattering is predominant (smaller angles), this is also according to Mie scattering theory, for particles much bigger than the wavelength of the incident light, as presented in Section 3.

To be able to mathematically determine the effect of light extinction, we need to investigate both phenomena, scattering and absorption. Scattering means, in fact, the change of the direction of transmission of the incident photon, but without any change in photon’s or particle’s energy. On the other hand, absorption means that the part of the photon’s energy is transferred to the particle. Here, the impact of the particle size is backward when comparing to scattering and the absorption cross-section is proportional to the particle’s projected area. Of course, besides the size, the particle’s shape and composition influence the level of absorption. What was previously presented in this section refers to scattering and absorption that are caused by a single particle, but, in a real environment, we have to deal with collections of very many particles. Inside a fog cloud, there is a continuous dynamic, particles are melting, which can lead to a change of their absorption coefficient [29]. The extinction is composed of scattering and attenuation (see Section 3) with all of the particles parameters mentioned before.

The way that we choose to investigate the effect of the fog particles on the laser beam is by taking slices from the beam, with the thickness equal to the particle diameter (will be used the average value considering the statement that the absorption cross-section is proportional with the particle projected area [29]) and approximate the effect of a slice on the incident light; afterward, we will extrapolate to the entire laser trace.

**Figure 7:**
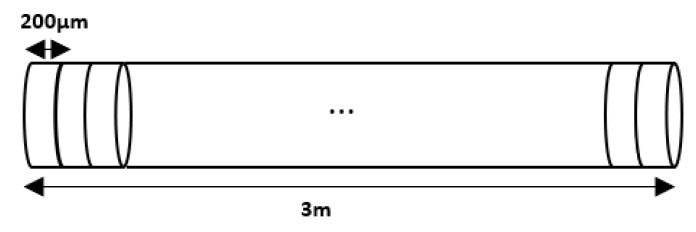
Impact of every slice from the laser beam.

We assumed the laser beam diameter being $D_{beam}$ = 1 mm; this leads to an area $A_{beam}$ = 0.785 mm, which gets in contact with the fog particles. If we are looking now to the first example presented in this section and we extrapolate the number of particles to the actual beam’s area, we will get a number of 778 particles for the actual surface; going deeper into the analysis, we will treat this surfaces as sections from the beam, with the thickness being equal to the average particle diameter ( 199 μm), as is figured below (Figure 7); this particle dimension is not proper for a fog particle, but, in this experiment, we planned to present the principles of how visibility is influenced by such atmospheric particles. In this case, the section’s volume will be $V_{sect}$ = 0.156 m$m^{3}$. The total length that we want to analyze is 3 m, the length of our experimental setup, which leads to a number of 15,075 slices and results in a total volume $V_{beam}$ = 2352 m$m^{3}$.

To be able to create a link between the impact of fog particles on incident light and visual acuity, we will use the first value from Section 4.2 (which will be further presented) for LWC (liquid water content), 0.87 g/$m^{3}$ (which has an initial impact of 78% decrease on optical power and reduces the visual acuity to the fourth row from the table with optotypes—caused by both effects, scattering and absorption, without having the possibility to split them). Using these data, we can calculate a mass of 2048 × $10^{-9}$ g fog particles inside the laser beam volume. Going backward, we can note that there are 135.85 × $10^{-12}$ g per slice and the total mass of an average particle is 0.17 pg (pico gram).

Related to the intensity extinction caused by every slice (we will consider the first slice here), we can use Beer–Lambert law:

(11) $$I_{S1}=I_{0}e^{-k*t}$$

where $I_{0}$ is the intensity of the incident light, k is the extinction coefficient (being the sum between the absorption and the scattering coefficients *k* = α + σ), and t is the thickness of the slice.

(12) $$\ln\left(I_{S1}/I_{0}\right)=-k*t\to k=-\ln\left(I/I_{0}\right)/t$$

However, the output intensity after passing just a slice ($I_{S1}$) is unknown; we have the information that is related to the overall attenuation caused by absorption and scattering, so we can calculate the attenuation coefficient (μ) while using the same formula, but for the entire laser trace (*x* = 3 ×$10^{6}$ μm) this time:

(13) $$\mu =-\ln\left(I_{out}/I_{0}\right)/x\to \mu =0.4*10^{-6}$$

Based on this information, we can estimate an average extinction coefficient per slice:

(14) $$k=\mu /15075=0.265*10^{-12}$$

It can be concluded that the amplitude of a scattered ray, at a certain scattering angle, is dependent on the divergence factor, attenuation factor, and the total phase shifts. In other words, in calculating the attenuation of a light beam, in addition to its wavelength, the size of the particles encountered in the path, the number (density) of particles in the path and the angle at which the wave intersects a particular particle must be taken into account; these conditions are difficult to control in a dynamic environment such as a fog cloud. Due to this reason, the method of analyzing the influence of just a section from the laser beam’s path on the incident light, can offer mathematical results close to the empirical ones. In Section 4.2, these relations will be used to create a link between the mathematical part and experimental part.

### 4.2. Visual Acuity—Measurement Systems vs. Observers

**Figure 8:**
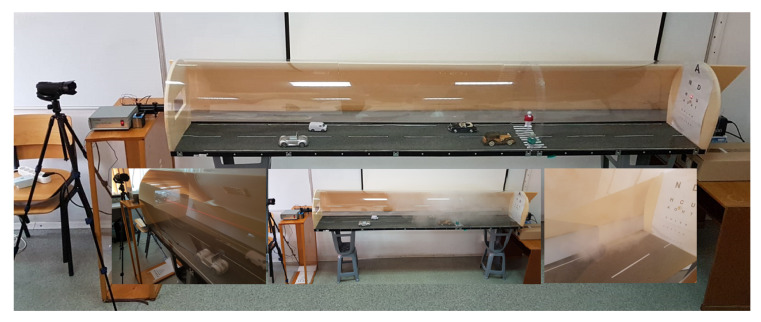
Laboratory setup.

The experimental setup realized in the laboratory, as presented in Figure 8, is three meters long and it has a volume of 0.576 $m^{3}$. This was realized to be able to create a foggy environment inside it. To create fog, we used the fog generator described in Section 4.1. During the experiments, the fog liquid is monitored using a balance (with an accuracy of 0.1 g) to calculate the fog density created inside the chamber. Visual acuity was measured in different foggy conditions using an automatic system (OCR algorithms), but also with human observers. Figure 8 presents, besides the overall structure (top), a zoom in the setup from the area where the laser source transmits the light beam and the camera monitors the decrease in visual acuity (left), the setup with fog inside it (center) and a zoom in the area with the optical receiver and the chart with optotypes (which are useful in monitoring the visual acuity) (right).

The preconditions for the experiments were: temperature 20 ${}^{\circ }C$, 400 Lx (±10 Lx) for daytime conditions, and under 20 Lx for night conditions; the values can be different on a sunny or cloudy day, but in this experiment, the main goal is to present some principles. Regarding the visibility, the systems and the observes (without eye diseases) were able to read the seventh row from the optotypes, which means normal visual acuity.

For the first experiment, we used 0.5 g (+/− 0.1 g) liquid to generate fog, which means that at a volume of 0.576 m${}^{3}$ the fog density, inside the chamber was 0.87 g/$m^{3}$. In real conditions, the typical values for liquid water content (LWC) are 0.05 g/$m^{3}$ for medium fog and 0.5 g/$m^{3}$ for dense fog, but having a short distance of measurement, we needed to increase the values. Before generating fog, the measured output power (at a distance of 3 m) was 1400 μW and observers were able to read the seventh row (R7) from the eye chart, which means normal visual acuity. If a person can read the optotypes from the sixth row (R6) of the eye chart means that the object to be observed needs to be twice as big than for a normal visual acuity while the first row (R1) of optotypes means that the object to be observed has to be ten times larger than for a normal visual acuity.

**Figure 9:**
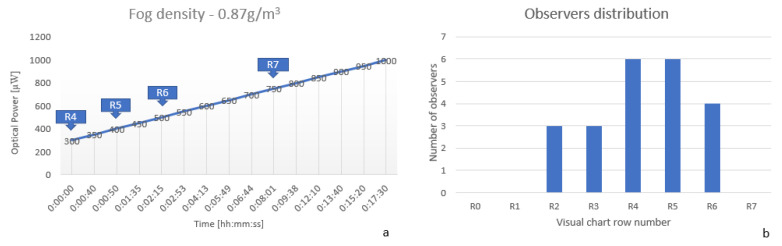
(**a**) Optical power and Optical Character Recognition (OCR) measurements at a fog density of 0.87 g/$m^{3}$ (**b**) Observers visual acuity distribution at a fog density of 0.87 g/$m^{3}$.

After generating the fog and waiting for 30 s for homogenization inside the chamber, the measured output power decreased at 300 mW, and the row that was still visible by the camera (using an OCR algorithm) was row number 4 (noted R4 on Figure 9a). One minute later, the output power increased to 400 mW, and on the eye chart was visible the 5th row. As the time elapsed, the visibility improved, and this happened due to collision coalescence/fusion of the fog particles. The basis theory shows that, due to the Brownian movement, turbulence and gravity influence particle collision takes place. Visibility is more impacted when there is a bigger density of smaller particles, due to their movement in the environment. In [30], Yu et al. realized an analysis of the drop radius increase while time elapses; the growth rate is pretty slow from 1 to 8 μm, but, afterward, the rate of growth increases rapidly. Due to the statements that are listed above, we tried to limit the monitoring time for every group of observers under five minutes, so that the fog conditions to be approximately the same.

The experimental conditions were reproduced, but this time the evaluation was done with 22 human observers, split into two groups, in order to keep as much as possible, the same visibility conditions. For Figure 9b, there is no elapse time highlighted for the identification of the optotype by human observers, because we considered that the visibility test is performed in less than five minutes, an interval that does not lead to a significant change in the experimental conditions. As mentioned before, we split the observers in two groups and the evaluation time was narrowed, so the error tolerance was minimized. The results can be observed in the figure above (Figure 9b), where most of the observers (73%) offered results in the same interval like the automatic system; for 27% of the observers, the results were weaker than the ones obtained with the automatic system, but more than half from these observers had known eye diseases and were not wearing the glasses during the experiments; we can already see a high risk of driving on public roads in these foggy conditions, 12 of the 22 observers not being able to observe at least row 5 of the table with optotypes, considered as a threshold of visual acuity to drive on public roads.

**Figure 10:**
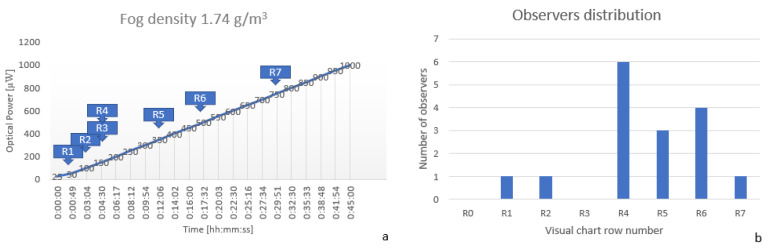
(**a**) Optical power and OCR measurements at a fog density of 1.74 g/$m^{3}$ (**b**) Observers visual acuity distribution at a fog density of 1.74 g/$m^{3}$.

For the second experiment, we used 1 g (±0.1 g) liquid to generate fog, so the fog density inside the chamber was 1.74 g/$m^{3}$. All of the experiments were monitored until the output optical power reached 1000 mW. In Figure 10a can be observed that in the first moments after fog was generated, visibility was close to zero but after around four minutes it increased significantly. Most of the period, the visual acuity spanned between the fourth and sixth row. Looking now at Figure 10b, it can be observed that most of the observers (13 out of 16) obtained results between the fourth and sixth, so the results are pretty much the same between the automatic system and observers. The slope of optotypes detection was similar to the one with the laser measurement system.

**Figure 11:**
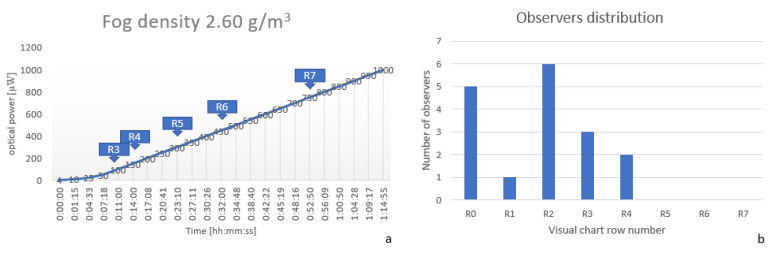
(**a**) Optical power and OCR measurements at a fog density of 2.60 g/$m^{3}$ (**b**) Observers visual acuity distribution at a fog density of 2.60 g/$m^{3}$.

In the third experiment, the quantity of liquid that was used for fog generation was increased to 1.5 g (+/−0.1 g), meaning that the fog density inside the chamber was 2.6 g/$m^{3}$. In Figure 11a can be observed that in the first 10 min visibility was really weak, under the third row. For the observers, the behavior was the same, from the first seven observed five were not able to see anything. After a few minutes, the visibility became better, which can be observed in both images Figure 11a,b. When comparing with the previous two experiments, for this fog density the visual acuity is really weak, and driving is not recommended in such visibility conditions. All of the observers are below the visual acuity threshold defined as mandatory to be reached in order to drive on public roads (row 5 of the optotype table).

**Figure 12:**
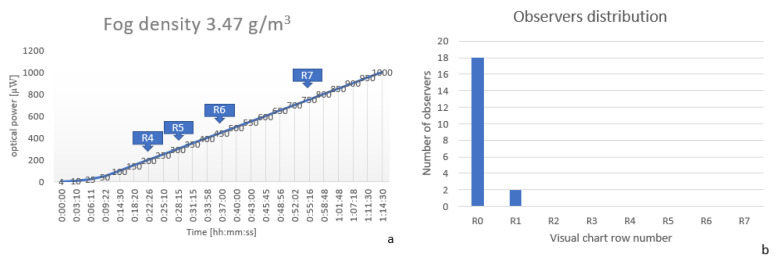
(**a**) Optical power and OCR measurements at a fog density of 3.47 g/$m^{3}$ (**b**) Observers visual acuity distribution at a fog density of 3.47 g/$m^{3}$.

In the last experiment, fog density was increased to 3.47 g/$m^{3}$ (2 g of liquid used). The visibility was zero for a long period of time, as confirmed by the system but also by the observers, 18 out of 20 were not able, in the first ten minutes, to discern any optotype from the eye chart, while the other two observers could see the first optotype (see Figure 12).

Table 1 presents a summary of the above-mentioned cases, realizing a link between the fog density, the decrease of the optical power and the visual acuity reported by the system respectively by the human observers. In this table are captured the values reported by the system at around 60 s after generating fog inside the chamber, while, for the human observers, are listed the most predominant values that were obtained after the experiment.

After these experiments can be concluded that at a fog density bigger than 0.87 g/$m^{3}$, driving on public roads is not recommended, and this statement is supported by both categories, system and human observers.

In order to link the mathematical results (obtained in Section 4.1) with the experimental one from this subsection, we will treat a specific case, the moment when the fifth row of optotypes is visible, with this one being considered the minimum visibility requirement to be able to drive on public roads, according to the European regulations. Accordingly, for every fog density considered in the experiments from Section 4.1, we calculated the total attenuation coefficient μ and an average slice attenuation coefficient α, at the moment when the fifth row was visible. These means that, even if in the initial moment when fog was generated, the fog particles’ mass inside the laser beam was different, after a specific time *t*, this becomes similar for each fog density, and this happens due to the particle collision (Brownian movement, turbulence, and gravity). Based on all of these data, we propose a model of identifying an optical power attenuation at a specific distance, by extrapolating the values obtained in the laboratory for a distance of three meters, to a bigger distance. The total attenuation coefficient at a specific distance d${}_{x}$(μd${}_{x}$), can be calculated by multiplying the mean attenuation coefficient of a slice ($\alpha_{mean}$, which can be considered 0.3 × $10^{-12}$) with the number of slices of the desired observation distance (calculated as d${}_{x}$/D${}_{ap}$, D${}_{ap}$ being the diameter of an average fog particle, 199 μm for the fog generated in the laboratory). Going further, we can also estimate the visibility distance, based on the theory of designing an eye chart—the observation distance shall be 68.75 times bigger than the biggest optotype from the chart. As mentioned before, our target is the 5th row, this one being five times smaller than the biggest optotype from the chart; this leads us to the statement than the observation distance is 13.75 times bigger than the object to be observed (e.g., road signs, being of standard dimensions). The principles that are mentioned above and summarized in Table 2 are very useful for a stationary system, installed near a highway or express road, knowing the distance between the equipment and having the main goal of estimating the weather conditions, estimating a visibility distance and notifying the drivers.

### 4.3. Lidar and Telemeter Measurements

**Figure 13:**
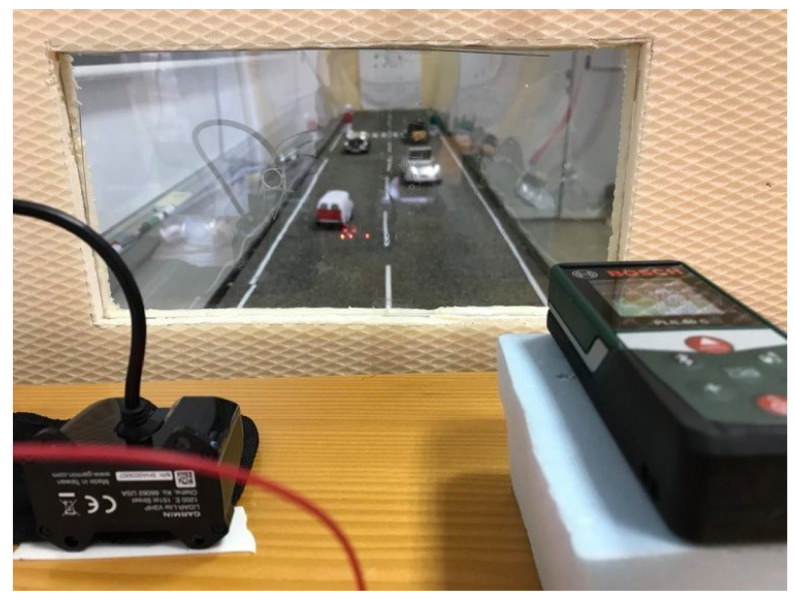
Lidar and Telemeter.

During the previous experiment, we measured the fog impact on direct transmission, in this subsection, we analyzed the impact on backscattering. For this, we used two devices, a LIDAR, and a Telemeter/laser rangefinder (Figure 13). The LIDAR device is a Lite V3hp model, controlled by an Arduino, which offers a sampling rate of 1kHz and is able to realize measurements between 5 cm and 40 m with an accuracy of ±2 cm for distances greater than 2 m; it works on a wavelength of 905 nm. The Telemeter is a Bosch PLR 40 c model, which is able to measure distances up to 40 m with an accuracy of ±2 mm, the measurement time being under 0.5 s; it works on a wavelength of 632 nm. During the experiments, we reproduced the fog conditions from Section 4.2 in order to have a linkage between the results.

The results obtained in five different fog conditions, by repeating five times every condition, are displayed in Table 3. We started the measurements in no fog conditions, and we adjust their positions in such a way to have the same values returned for the distance (3 m). After generating fog from 0.5 g liquid, the results that are offered by the telemeter are unchanged, while the distance offered by the LIDAR decreases. For 1 g of liquid ( 1.74 g/$m^{3}$ fog density inside the chamber) the telemeter is still not influenced whereas the LIDAR measures a distance of 1.27 m, which means a decrease of almost 60%. Increasing the fog density to 2.6 g/$m^{3}$ means an impossibility of measurement for the telemeter while the LIDAR still measures but the distance is smaller, 0.86 m. For the last fog conditions, when we used 2 g of liquid for generating the fog, the telemeter remained in the same state (error in measurement), while the LIDAR still measures a distance, but it is strongly influenced by fog particles.

The behavior of the two devices in fog conditions is quite interesting, the telemeter offers correct results until a specific fog density and then follows a hard cut when the device displays error in measurement or measures the distance till the fog cloud (10 cm—see Figure 13); on the other hand, the LIDAR is impacted from the first moment when fog is present in the setup but it is still able to perform some measurements in fog and reduces the return measured value while the density of fog particle increases.

### 4.4. Safety System for Visibility Distance Estimation

Our system proposal for visibility measurement is a combination of sensors—a stationary one that measures direct the optic power attenuations (for laser radiation) and is installed near the public roads and a lidar installed on vehicles. The synchronization of these systems will increase traffic safety in bad weather conditions, the efficiency being better, since the results are gathered from several systems (stationary and moving). The stationary system will measure the fog density that is based on the decrease of optical power and will estimate a visibility distance; afterward the results are sent to the vehicle systems via RF radios. The lidar device (placed on the vehicle) will detect possible objects in front of the vehicle but will also estimate visibility in case of fog conditions. Gathering the information from all of these sensors the vehicle system can notify the drivers about possible dangers and can take actions, like reducing the speed or automatic stop in case of danger.

**Figure 14:**
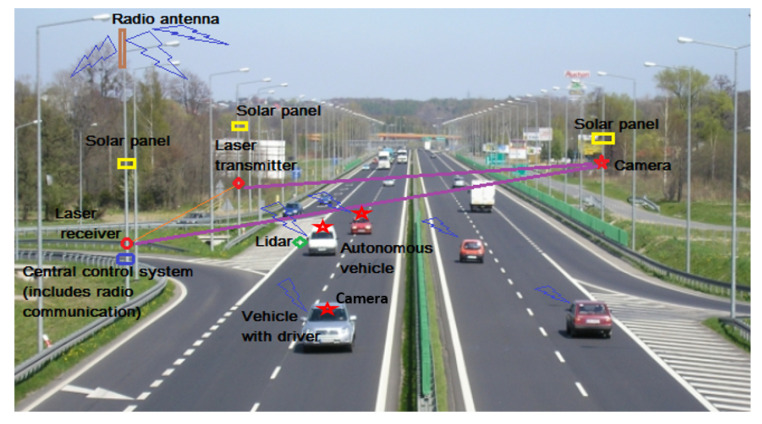
Proposal for a system to increase traffic safety in real traffic conditions.

Figure 14 shows a real traffic situation on the highway, highlighting the system elements that ensure safety in low visibility conditions: laser transmitters, optical receivers, and cameras for stationary systems, respectively, lidar and camera for mobile systems. In the near future, there will be more and more frequent cases in which autonomous cars will appear on public roads alongside the classic ones driven by drivers (humans). As drivers’ actions are unpredictable, these systems will have to have an extremely short evaluation and reaction time in order to be able to avoid possible accidents. The stationary model is installed near the roads (parallel to them, in the travelling direction) in order not to impact traffic participants, but also to avoid interposing obstacles between the transmitter and the optical power measurement sensor; for this system, there is no risk to causing eye issues. The power supply of the stationary systems’ components can be made locally with batteries and solar panels. The lidar device is already installed on current vehicles, a fact that guarantees it was certified against causing eye issues; in this case, the lidar can have a new functionality, that of detecting weather conditions, and together with the stationary system to form a much more efficient collaborative system.

The results of the information processing (notifications) are transmitted by means of a radio transmitter to the vehicles (ECUs) located in the respective area and the highway’s display panels (if they are available in the area). On board the vehicles, the results will be compared with those that are offered by their own systems (mounted on board, such as LIDAR) and the corresponding decisions will be made.

## 5. Discussion and Conclusions

Visibility is a critical aspect of transportation and fog is one of the biggest enemies for visibility on public roads. Unfortunately, this issue is not solved, even by the autonomous vehicles; another big trend from the automotive industry, besides autonomous vehicle, is connectivity with the well-known V2V or V2X concepts but even so bad visibility that is caused by extreme weather conditions can cause fatalities (e.g., pedestrian not observed, road marking missed by the vehicles, vehicles not part from the network, or other objects on the public roads not observed, etc.).

The goal of our work is to present a generic collaborative systems that can be used on public ways and help the drivers or the autonomous driving system in bad weather conditions. Our personal contributions in this paper are:

- We managed to realize a laboratory setup where we succeeded to test different fog detection (and visibility estimation) methods in similar and repeatable conditions;

This gave us a big advantage in understanding the fog effect, because, in real conditions, it is hard or almost impossible to have two times the same fog conditions to be able to validate the results. We compared the results got with the measurement systems (based on laser or LIDAR) with the results obtained from human observers and we concluded that are in the same range, differences appeared for observers with eye diseases.

**Figure 15:**
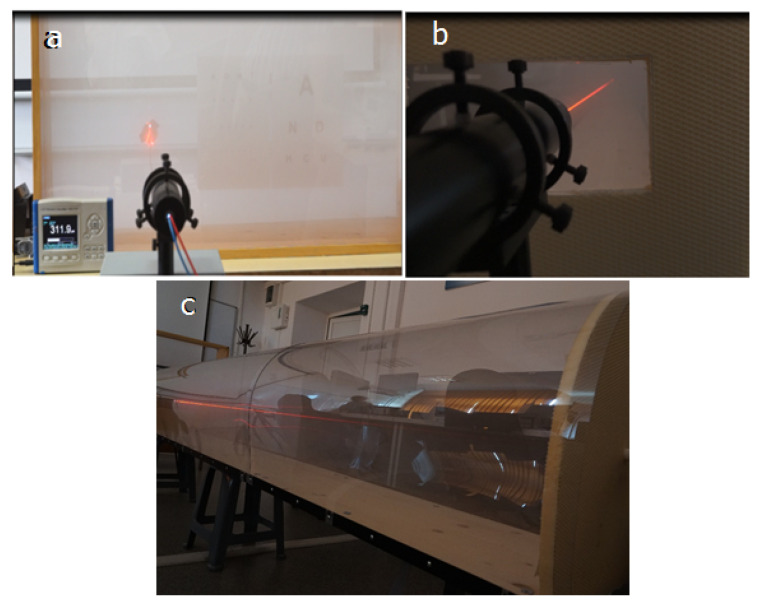
Impact on laser beam for (**a**). Fog layer of 30 cm (**b**) and (**c**). Uniform fog of 3 m (seen from two positions).

In the measurement phase, we did two sets of experiments: one when we created a dense fog layer of 30 cm Figure 15a) and one in which the same amount of fog is spread on the length of 3 m (Figure 15b,c). In the second case, the measurements were made when the fog became uniform (30 s after generating it).

With these two experimental models (Figure 15) the connection/correlation can be identified between the decrease of the optical power and the decrease of the visual acuity in fog conditions. The measurements were made in both cases from 3 m, the difference being that for the first case only a layer of fog of 30 cm is created and, in the second case, the fog is constant/homogeneous (although it is difficult to say that it can be 100% homogeneous, because it is made up of waves of particles, it does not have a unitary structure) throughout the 3 m. This length, 3 m, is the distance that is used by ophthalmologists when assessing visual acuity using optotype tables.

In Table 4 and Table 5, parallels are presented between the fog impact on optical power decrease and on visual acuity for different amounts of fog, for the two cases mentioned above. These results lead us to the conclusion that the laser beam is impacted by the number of particles encountered on its path (disperse over a length or in a limited space, but with higher density). It allows us to perform experiments at the laboratory level and extrapolate the resulting principles to real conditions.

The measurements were performed in uniform fog, as mentioned in Section 3.4 “after around 30 s (time need to have a uniform fog spread inside the chamber)”. In Figure 15c can also be seen how the laser beam from the transmitter to the receiver is attenuated. In Figure 8, the left side, it can also be observed that fog is uniform inside the setup, for the center and right pictures there were moments when fog was generated at that time.

- We presented the fog effect on direct transmission and on backscattering;

For the first category, we identified fog densities where driving is not recommended and densities were driving is forbidden; for the latter category, we used two devices, a LIDAR, and a telemeter, in order to highlight their different behaviors in fog conditions. The lidar device proved to be more efficient in fog environment, being able to detect fog and highlight a decrease of visibility.

- We presented a way in which a visibility distance can be estimated from an optical power measurement (link between the loss of optical power in different fog conditions and the visual acuity of human observers);

Let us consider two examples for the first category, while using the results that are presented in Table 2:

- The dimension of a road sign is 70 cm, so at an attenuation of around 75% of the input optical power (measured after passing the fog cloud), it is visible from a distance of around 50 m (considering the biggest optotype in the determination) while from a distance of around 10 m, the smallest details are noticeable (when considering the fifth row of optotypes).
- A pedestrian of 1.7 m high, in the same fog conditions described above, is visible from a distance of around 115 m, the smallest details being distinguishable from around 23 m. Based on this distance (estimated using optical power measurements and applying some mathematical algorithms) and when considering the vehicle type can be recommended a safety travel speed, using the following relationship [31]:

(15) $$v=\left(-t_{r}+\sqrt{t_{r}^{2}+\frac{2s}{g\left(f+G\right)}}\right)*\left(g\left(f+G\right)\right)$$

The recommended speed can be determined using some additional information like the driver’s reaction, the coefficient of braking friction or the slope of the road, where:

*v* = the car’s speed (m/s)

*s* = the distance in which the car can be stopped (m)

*t${}_{r}$* = the driver’s reaction time (sec)

*g* = acceleration due to gravity (m/s${}^{2}$)

*f* = coefficient of braking friction

*G* = gradient (slope of the road) 

Of course, the results that are offered by the stationary system shall be compared with the ones offered by the mobile systems and afterwards the vehicle’s main system can take a decision.

- Using the experimental set up described in our work, the methods that are based on image processing can also be tested in real time and under repeatable conditions.

For Intelligent Transportation Systems, the use of cameras is essential for the recognition of various scenes (traffic signs, road markings, pedestrian etc.) The demonstrator presented in the paper was created to allow us to test also image processing methods, but this part was not considered in the current paper. We refer to both groups of methods: of detecting fog (like the stationary systems installed close to the public roads—see Figure 14), as well as to those of improving visibility or estimating visibility distance in fog conditions (the mobile ones installed on the vehicles—see Figure 14). No such tests can be performed in the natural environment in order to validate the applicability and the correctness of the results of those methods.

The optical power measurements methods shall be much faster than the image processing methods, related to the task of fog detection and visibility estimation, but these aspects need to be tested and confirmed.

The validation of the collaborative system in real conditions requires authorizations to be installed on highways/public roads and adapted vehicles to the collaboration with the fixed distance evaluation system. All of these require additional costs and a rather long period of experimentation, conditioned by the periodicity of the fog phenomenon.

In this paper, the measurement principles were established and the results were obtained by experiments/measurements that were realized on a laboratory setup created by the authors.

## Figures and Tables

**Table 1:** Results for visual acuity measurement—system vs human observers.

Measurement Distance	Measurement Time	Liquid Quantity [g]	Fog Density [g/m${}^{3}$]	Optical Power [uW]	Visual Acuity System	Visual Acuity Human
3 m	60 s	0	-	1400	R7	R7
		0.5	0.87	420	R5	R5/R4
		1	1.74	60	R2	R4
		1.5	2.6	noise	-	R2
		2	3.47	noise	-	-

**Table 2:** Estimating visual acuity based on attenuation coefficient.

Generated Fog Density [g/m${}^{3}$]	Fog Particles Mass in Laser Beam [g]	Time Elapsed, *t* [min:sec]	Visual Acuity	Total Attenuation Coefficient, μ	Attenuation Coefficient/Slice, α	Extrapolate Attenuation to a Specific Distance, d${}_{x}$	Extrapolate Visual Acuity to a Specific Distance, d${}_{x}$
0.87	2048 × $10^{-9}$	0:50		0.418 × $10^{-6}$	0.277 × $10^{-12}$		
1.74	4097 × $10^{-9}$	12:06	R5	0.462 × $10^{-6}$	0.306 × $10^{-12}$	μd${}_{x}$ = $\alpha_{mean}$	d${}_{x}$ = 13.75
2.6	6132 × $10^{-9}$	23:10		0.513 × $10^{-6}$	0.340 × $10^{-12}$	× (d${}_{x}$/d${}_{ap}$)	× object dim
3.47	8171 × $10^{-9}$	25:15		0.513 × $10^{-6}$	0.340 × $10^{-12}$		

**Table 3:** The results for LIDAR and Telemeter in different fog conditions.

Measurement Distance [m]	Liquid Quantity [g]	Fog Density [g/m${}^{3}$]	Lidar Results [m]	Telemeter Results [m]
3	0	-	3	3
3	0.5	0.87	2.86	3
3	1	1.74	1.27	3
3	1.5	2.6	0.86	Error
3	2	3.47	0.64	Error

**Table 4:** Parallel between loss of optical power and visual acuity for different amounts of fog in a layer of fog of 30 cm and observation of the table from 3 m.

Measurement Distance	Visual Acuity	Optical Power [uW]	Liquid Quantity [g]	Fog Density [g/m${}^{3}$]
3 m Fog layer thickness = 30 cm	0	57	2	11.11
	1	120	1.75	9.72
	2	140	1.5	8.34
	3	220	1.25	6.95
	4	280	1	5.56
	5	340	0.75	4.17
	6	550	0.5	2.78
	7	950	0.25	1.39

**Table 5:** Parallel between loss of optical power and visual acuity for different amounts of fog for uniform fog and observation of the 3 m table.

Measurement Distance	Visual Acuity	Optical Power [uW]	Liquid Quantity [g]	Fog Density [g/m${}^{3}$]
3 m Fog layer thickness = 30 cm	0	57	2	3.47
	1	120	1.75	3.05
	2	140	1.5	2.60
	3	220	1.25	2.14
	4	280	1	1.74
	5	340	0.75	1.31
	6	550	0.5	0.87
	7	900	0.25	0.44
